# Sympathetic neurons are a powerful driver of myocyte function in cardiovascular disease

**DOI:** 10.1038/srep38898

**Published:** 2016-12-14

**Authors:** Hege E. Larsen, Konstantinos Lefkimmiatis, David J. Paterson

**Affiliations:** 1 OXION Initiative in Ion Channels and Disease, Sherrington Building, University of Oxford, Oxford OX1 3PT, UK; 2 Burdon Sanderson Cardiac Science Centre, Department of Physiology, Anatomy and Genetics, University of Oxford, UK

## Abstract

Many therapeutic interventions in disease states of heightened cardiac sympathetic activity are targeted to the myocytes. However, emerging clinical data highlights a dominant role in disease progression by the neurons themselves. Here we describe a novel experimental model of the peripheral neuro-cardiac axis to study the neuron’s ability to drive a myocyte cAMP phenotype. We employed a co-culture of neonatal ventricular myocytes and sympathetic stellate neurons from normal (WKY) and pro-hypertensive (SHR) rats that are sympathetically hyper-responsive and measured nicotine evoked cAMP responses in the myocytes using a fourth generation FRET cAMP sensor. We demonstrated the dominant role of neurons in driving the myocyte ß-adrenergic phenotype, where SHR cultures elicited heightened myocyte cAMP responses during neural activation. Moreover, cross-culturing healthy neurons onto diseased myocytes rescued the diseased cAMP response of the myocyte. Conversely, healthy myocytes developed a diseased cAMP response if diseased neurons were introduced. Our results provide evidence for a dominant role played by the neuron in driving the adrenergic phenotype seen in cardiovascular disease. We also highlight the potential of using healthy neurons to turn down the gain of neurotransmission, akin to a smart pre-synaptic ß-blocker.

Many therapeutic interventions in states of heightened adrenergic activity associated with cardiovascular disease are targeted at the myocyte, suggesting these cells are of primary importance in driving the disease process^1^,^2^. However, emerging clinical evidence suggests that removal of sympathetic nerves that innervate the heart (cardiac stellectomy) improves morbidity and mortality caused by arrhythmias and sudden cardiac death^3^, although the ability of the neuron to drive the myocyte phenotype in disease has not been firmly established. Nevertheless cardiac sympathetic hyperactivity is a well established early hallmark of heart failure^4^,^5^, post myocardial infarction^6^ and hypertension, both in humans^7^,^8^,^9^,^10^,^11^ and in the spontaneously hypertensive rat (SHR)^12^,^13^. In the pro-hypertensive SHR, the sympathetic stellate neurons that predominantly innervate the heart^14^ show increased membrane Ca^2+^ currents^15^,^16^, intracellular Ca^2+^ transients^17^ and significant impairment of the noradrenaline reuptake transporter (NET)^18^ that all contribute to enhanced noradrenaline (NA) release^13^,^19^,^20^. This heightened sympathetic activity at the end organ results in ß-adrenergic hyper-responsiveness of the myocyte^21^,^22^,^23^. In addition, sino-atrial cells^21^ and ventricular myocytes^22^ from the SHR also display increased basal and stimulated heart rate^13^,^19^, linked to significantly greater basal and NA stimulated L-type Ca^2+^ currents^21^,^22^.

Since the disease phenotype resides in two spatial domains, the relative contribution each makes to dysautonomia remains unclear, even though the (patho-) physiology of the individual ion channels and signalling molecules on the single neuron and single myocytes as separate systems are well described. Surprisingly relatively little is known about the cell-to-cell interaction that takes place in disease^24^,^25^. What is now becoming clear is that sympathetic neurons play a larger role in modulating the behaviour of myocytes than previously thought^8^,^26^,^27^. This occurs both via anterograde signalling^28^, but also through eliciting changes in the expression of ion channels and receptors on the myocyte membrane that are critically involved in myocyte function^29^,^30^,^31^, and more recently, through changes in the structure of the neuro-cardiac junction^31^,^32^. To fully understand the importance of sympathetic neurotransmission on end-organ function and how it might be altered in disease^28^, we developed a model system to study the peripheral neuro-cardiac axis itself, rather than the cells in isolation. We also used a novel cAMP FRET (Förster Resonance Energy Transfer) sensor to measure post synaptic sympathetic drive when we cross-cultured normal neurons onto diseased myocytes and vice versa as we attempted to modulate the myocyte phenotype.

Here we addressed the following questions: (i) does sympathetic hyperactivity observed in the single neuron and single myocyte, translate into a native co-culture of functionally coupled sympathetic stellate neurons and ventricular myocytes? (ii) Is the neuron or the myocyte the primary driver of the cardiac adrenergic phenotype associated with the pro-hypertensive state?

## Results

### The co-culture phenotype and cross-culture formation

**Figure 1:**
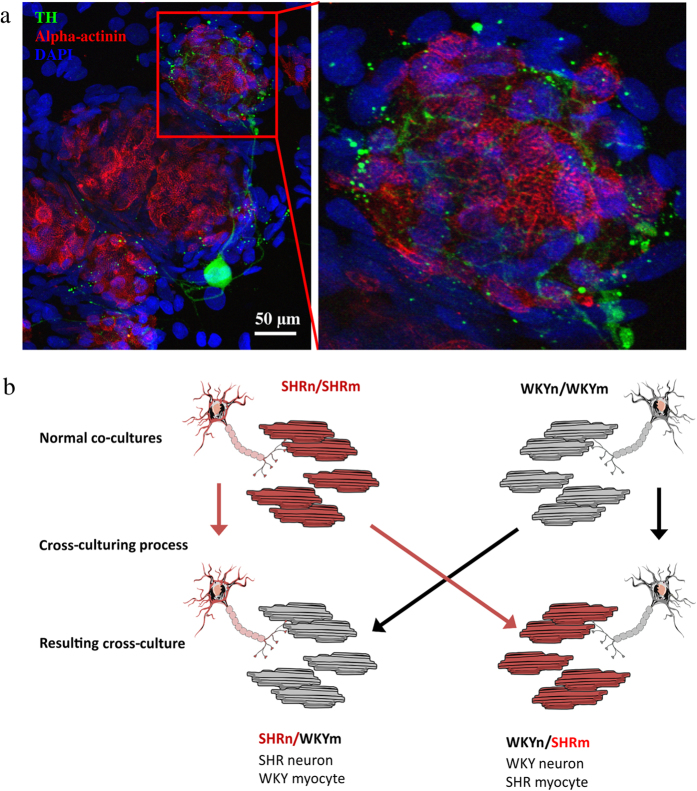
Co-cultures of ventricular myocytes and sympathetic stellate neurons. Cardiac sympathetic stellate neurons were plated on top of ventricular myocytes. Pro-hypertensive SHRnSHRm cultures were compared to normotensive WKYnWKYm cultures to test whether the sympathetic hyperactivity observed in the hypertensive state, translates into a co-culture model. Immunofluoresence staining confirmed the cellular phenotypes (**a**) and inset). Sarcomeric alpha actinin marks the myocytes (red), Tyrosine hydroxylase (TH – green) is a sympathetic neuron marker. The neuronal processes naturally interweave with the myocytes (inset) resulting in a rich innervation of the myocytes. To investigate which cell is the primary driver behind this cardiac autonomic phenotype, two cross-cultures were developed (**b**) and exposed to the same experimental protocols. Panel b was created using Servier Medical Art according to a Creative Commons Attribution 3.0 Unported License guidelines 3.0 (https://creativecommons.org/licenses/by/3.0/). Simplification and colour changes were made to the original neuron and myocyte cartoons.

The myocytes were densely innervated by the sympathetic neurons (Fig. 1a), analogous to that observed *in vivo*^33^. Immunofluorescence confirmed the cellular phenotypes with sarcomeric alpha-actinin denoting the myocytes, and tyrosine hydroxylase (TH) denoting the sympathetic neurons. The sympathetic neurons displayed the characteristic punctate staining of the varicosities, indicative of sites of synapse formation^28^,^34^ (Fig. 1a). Visual innervation was observed in all cultures that were recorded from. The cross-culture formation is detailed in Fig. 1b. The was no visual difference in the appearance of the WKYnSHRm or the SHRnWKYm when compared to the WKYnWKYm and SHRnSHRm (24, 42, 60 and 102 images respectively – data not shown).

### The myocytes from the pro-hypertensive SHR are over-responsive to beta adrenergic stimulation

**Figure 2:**
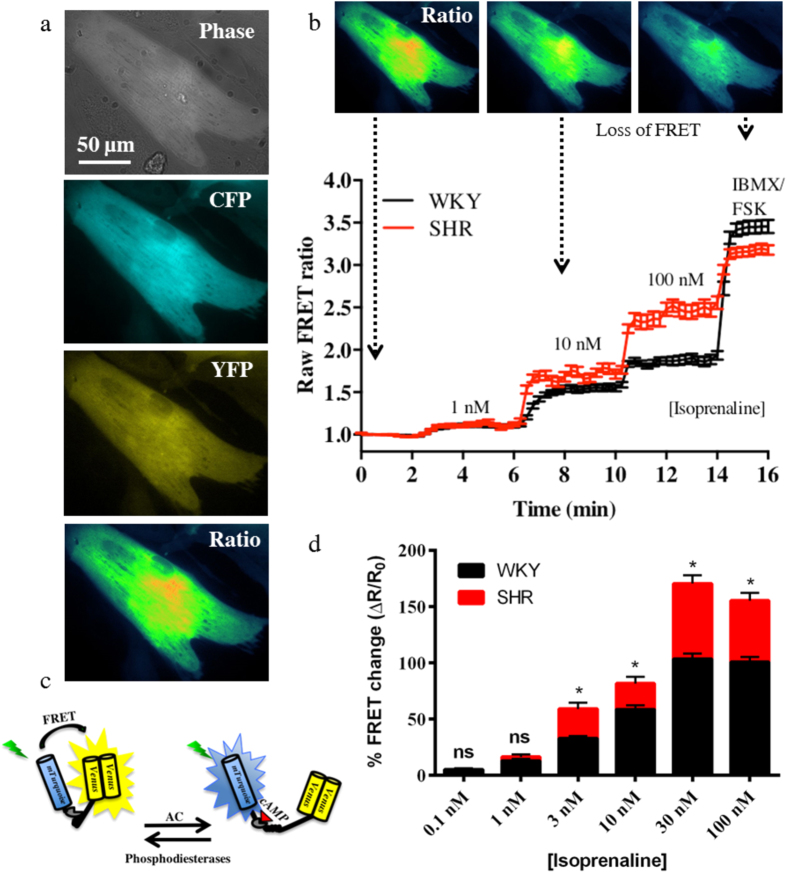
The ventricular myocytes from the pro-hypertensive SHR are hyper-responsive to β-adrenergic stimulation. To investigate the β-adrenergic responses of the myocytes, we perfused the cells with isoprenaline and used a fluorescence resonance energy transfer (FRET) sensor Ad-Epac-S^H187^ to detect changes in cAMP levels as a result of the stimulation. CFP and YFP fluorescence intensities were taken and the ratio plotted (**a**). The binding of cAMP to Ad-Epac-S^H187^ (**c**) causes a conformational change in the sensor that moves the fluorophores apart, thereby resulting in loss of FRET (**b**). The SHR myocytes had significantly larger cAMP responses to isoprenaline when compared to the WKY at concentrations ≥ 3 nM (**d**). Concentrations and n numbers (WKY:animals/coverslip/cells and SHR:animals/coverslips/cells): 0.1, 3, 30 nM (WKY:28/12/55 and SHR:7/6/33), 1, 10,100 nM (WKY:28/11/50 and SHR:7/5/27). *p < 0.001, two-way ANOVA with Bonferroni correction. The cartoon in panel c was modified from *Methods Mol. Biol.* Simultaneous assessment of cAMP signaling events in different cellular compartments using FRET-based reporters, **1294,** 2015, pp 1–12, Burdyga, A. & Lefkimmiatis, K. with permission of Springer^60^.

To test the ß-adrenergic responsiveness of the myocytes, we bath applied the ß-agonist isoprenaline and measured the changes in cAMP levels using FRET. Isoprenaline treatment of the myocytes revealed that the SHR myocytes were significantly hyper-responsive when compared to the WKY myocytes at isoprenaline concentrations ≥3 nM (Fig. 2). The responses increased over the concentrations tested and plateaued between 30 nM and 100 nM.

### The co-cultures from the pro-hypertensive SHR (SHRnSHRm) are hyper-responsive to nicotinic activation

**Figure 3:**
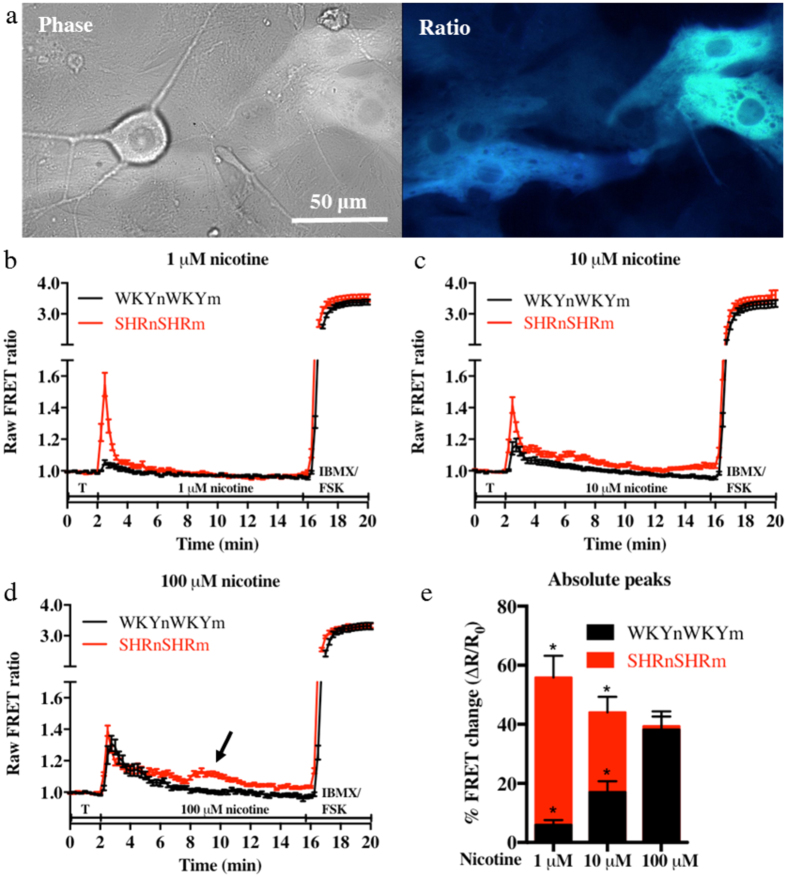
The co-cultures from the pro-hypertensive SHR animals are hyper-responsive when compared to the normotensive WKY. The myocytes of the pro-hypertensive SHR and WKY co-cultures were selectively infected with the Ad-Epac-S^H187^ cAMP FRET sensor (**a**). The neurons in the co-culture were stimulated using increasing concentrations of nicotine to stimulate sympathetic drive. The resulting effect of the released noradrenaline on the ß-adrenergic receptors was detected as changes in myocyte cAMP levels. The myocytes from the SHRnSHRm co-cultures had significantly higher cAMP responses to nicotine stimulation at 1 μM (55.89 ± 7.292%, n = 28 (13 animals/4 coverslips/28 cells) vs 5.952 ± 1.623%, n = 24 (29 animals/7 coverslips/24 cells), p < 0.0001: (**b**) and 10 μM (44.02 ± 5.310%, n = 36 (13 animals/4 coverslips/36 cells) vs 17.05 ± 3.715%, n = 29 (29 animals/9 coverslips/29 cells), p < 0.0002: (**c**) nicotine when compared to the WKYnWKYm cultures. At 100 μM nicotine the responses of the two cultures were the same (38.25 ± 6.105%, n = 20 (29 animals/7 coverslips/20 cells) vs 39.35 ± 3.283%, n = 27 (13 animals/3 coverslips/27 cells) p = 0.87: (**d**). b-d depicts the mean of all the raw data traces collected (±SEM). Statistics (unpaired t test) were performed on the absolute peak values expressed as % FRET increase from baseline (**e**).

Following the addition of nicotine to the co-culture, myocyte cAMP levels increased rapidly before returning to baseline confirming that the two cells were functionally connected. The myocytes from the pro-hypertensive SHRnSHRm co-cultures showed significantly larger nicotine-evoked cAMP responses when compared to the WKYnWKYm at 1 μM (55.89 ± 7.292%, n = 28 vs 5.952 ± 1.623%, n = 24, p < 0.0001) and 10 μM (44.02 ± 5.310%, n = 36 vs 17.05 ± 3.715%, n = 29, p < 0.0002) nicotine (see Fig. 3b,c). At 100 μM the responses of the two co-cultures were the same (38.25 ± 6.105%, n = 20 vs 39.35 ± 3.283%, n = 27, p = 0.87. Figure 3d). At the higher concentrations of nicotine (300 and 500 μM), significantly reduced responses were found in both co-cultures that were not significantly different from each other (results not shown). Both co-cultures responded identically to maximal activation of the sensor with 100 μM IBMX (a general phosphodiesterase - PDE - inhibitor) and 25 μM forskolin (WKY: 241.2 ± 5.94% n = 76 cells. SHR: 239.0 ± 5.74% n = 87 cells, p = 0.79). Furthermore, roughly half of the SHRnSHRm co-cultures portrayed double peaks (see arrow on Fig. 3d) of cAMP elevation. This double peak behaviour was not observed in any of the WKYnWKYm co-cultures.

### Myocyte cAMP responses in the co-cultures are mediated by the β_1_ adrenergic receptor.

**Figure 4:**
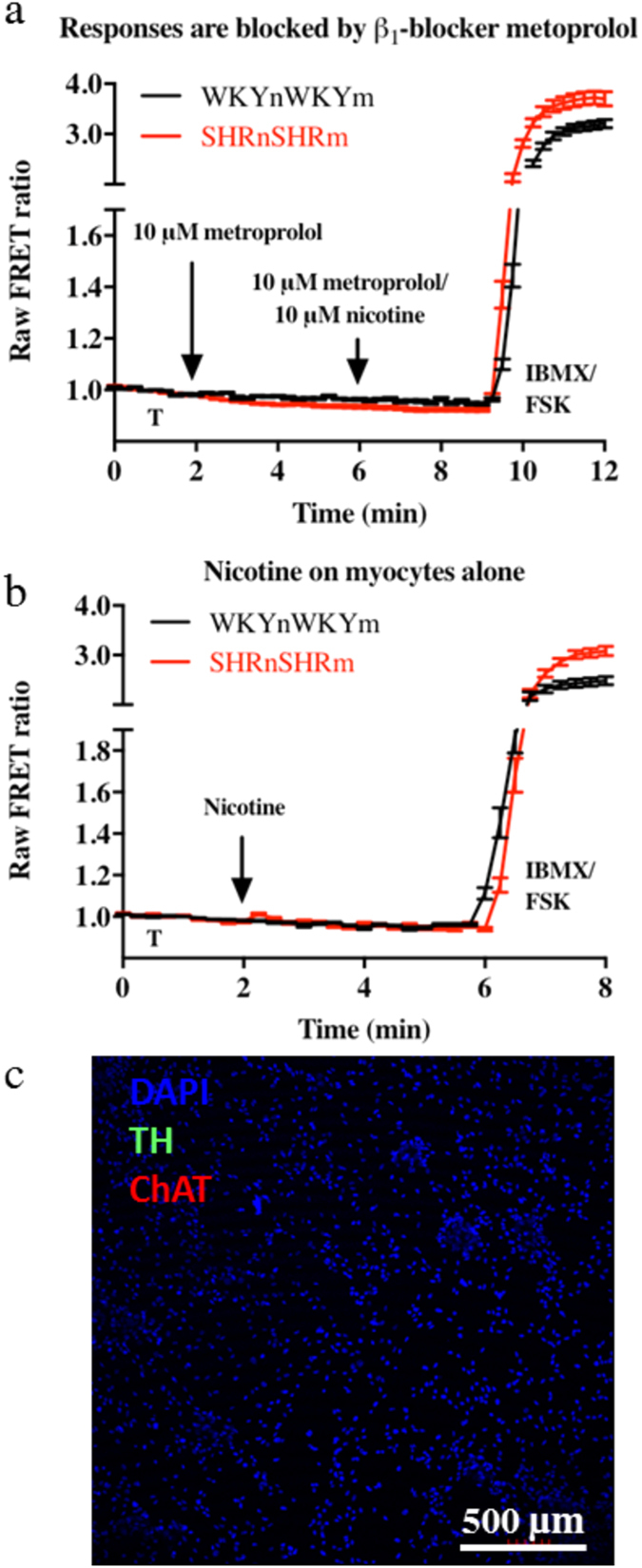
Myocyte cAMP responses are mediated by the β_1_ adrenergic receptor. Application of nicotine in the presence of the β_1_ selective antagonist metoprolol (10 μM) blocked all nicotine induced cAMP elevations in all cells tested (n = 60 cells) both in the WKY and SHR co-cultures (**a**). Nicotine application to the myocyte-only cultures (1–500 μM) had no effect on myocyte cAMP levels in all cells tested (WKY n = 9, SHR n = 20 cells) (**b**). Immunofluorescence was performed on each culture and showed negative Tyrosine hydroxylase (TH) and choline acetyltransferase (ChAT) stain conforming no contaminating neurons were present in the myocyte only cultures (**c**). This indirectly confirmed that the co-culture responses seen were solely due to sympathetic stellate neurons and not contaminating intra cardiac neurons. The data also confirmed that nicotine had no non-specific effect on cAMP levels in myocytes.

The application of the β_1_ selective antagonist metoprolol (10 μM) blocked all nicotine evoked cAMP responses (Fig. 4a) in the SHRnSHRm and WKYnWKYm co-cultures at all nicotine concentration and in all cells tested (n = 60 cells). To confirm the absence of contaminating intra-cardiac neurons in the myocytes culture, we tested the cAMP response to nicotine, and performed immunofluorescence on myocytes culture alone. Nicotine application (1–500 μM) to the myocytes did not change cAMP levels in any cells tested (WKY n = 9, SHR n = 20 cells) (Fig. 4b). Immunofluorescence staining for tyrosine hydroxylase (TH) and choline acetyltransferase (ChAT) confirmed the absence of sympathetic or parasympathetic intra-cardiac neurons in the myocytes cultured alone (Fig. 4c).

### Are the cardiac sympathetic stellate neurons the principle drivers behind the cardiac autonomic dysfunction associated with the pro-hypertensive state?

**Figure 5:**
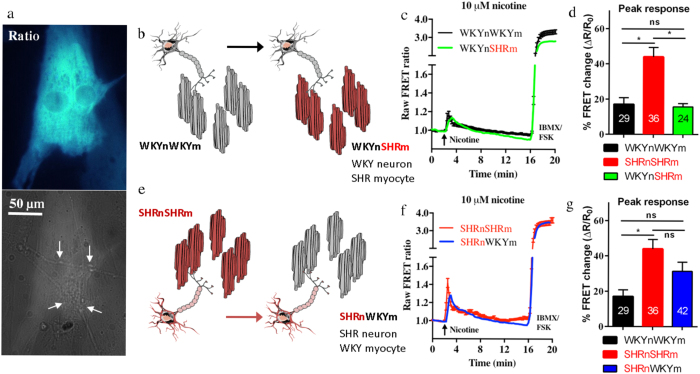
The cardiac sympathetic stellate neurons are the primary driver of the cardiac sympathetic hyper-activity associated with pro-hypertensive states. To investigate whether the neuron or the myocyte is the primary driver of the co-culture phenotype we see, we created two cross-cultures (see Fig. 1 for schematic). The myocytes were selectively infected with the cAMP Ad-Epac-S^H187^ FRET sensor (**a**) neuronal processes marked by the arrows before 10 μM nicotine was applied. The first cross-culture was created by plating healthy WKY neurons on top of pro-hypertensive myocytes, creating the WKYnSHRm cross-culture (**b**). The WKY neurons were found to attenuate the myocyte cAMP responses of the pro-hypertensive SHR myocytes (**c,d**). The nicotine evoked cAMP responses in the WKYnSHRm were significantly smaller than those of the SHRnSHRm (15.67 ± 1.936, n = 24 (9 animals/4 coverslips/24 cells) vs 44.02 ± 5.310, n = 36 (13 animals/4 coverslips/36 cells), p < 0.0001) and not significantly different from that of the WKYnWKYm cultures (15.67 ± 1.936, n = 24 vs 17.05 ± 3.715 n = 29 (29 animals/9 coverslips/29 cells), p = 0.76). Moreover, the pro-hypertensive SHR neurons were able to induce a diseased myocyte cAMP phenotype on the otherwise healthy WKY myocytes (**b,c**). The cAMP levels of the SHRnWKYm were substantially (though not significantly) larger than those of the WKYnWKYm (31.37 ± 5.194, n = 42 (21 animals/9 coverslips/42 cells) vs 17.05 ± 3.715, n = 29). This response was not significantly different from the diseased SHRnSHRm culture (31.37 ± 5.194, n = 42 vs 44.02 ± 5.310 n = 36, p = 0.094) suggesting a partial recapitulation of the diseased phenotype. (**c,f**) represent the mean raw traces of all recordings made. The mean without SEM is reported for the cross-cultures for clarity. Statistical analysis (one-way ANOVA with Bonferroni correction) was performed on the mean absolute peak data ± SEM (**d,g**). Panel b and e were created using Servier Medical Art according to a Creative Commons Attribution 3.0 Unported License guidelines 3.0 (https://creativecommons.org/licenses/by/3.0/). Simplification and colour changes were made to the original neuron and mycoyte cartoons.

To test the role played by the sympathetic neurons in the cardiac sympathetic hyper-activity, the nicotine-evoked cAMP responses of the cross-cultures (WKYnSHRm and SHRnWKYm were compared to the WKYnWKYm and SHRnSHRm co-cultures. We found that in the WKYnSHRm cross-cultures (Fig. 5b), the normal WKY neurons rescued the cAMP responses of the pro-hypertensive SHR myocytes (Fig. 5c,d). The nicotine-evoked cAMP responses of the myocytes in the WKYnSHRm were significantly smaller than those from the SHRnSHRm (15.67 ± 1.936%, n = 24 vs 44.02 ± 5.310%, n = 36, p < 0.0001) and not significantly different from that of the WKYnWKYm cultures (15.67 ± 1.936%, n = 24 vs 17.05 ± 3.715%, n = 29, p = 0.757). Further, in the SHRnWKYm cross-culture (Fig. 5e), the pro-hypertensive SHR neurons were able to induce induce a ‘diseased’ cAMP response in the otherwise healthy WKY myocytes (Fig. 5f,g). The nicotine-evoked cAMP responses of the myocytes from the SHRnWKYm culture were substantially (though not significantly) larger than those of the WKYnWKYm culture. However, this response was not statistically different from those of the diseased SHRnSHRm culture (31.37 ± 5.194%, n = 42 vs 44.02 ± 5.310%, n = 36, p = 0.094), indicating the diseased neurons had partially recapitulated the SHRnSHRm cAMP phenotype.

## Discussion

The novel findings presented here are: (i) the cardiac sympathetic dysfunction identified in the single diseased neuron and myocyte translates into coupled co-cultures; (ii) the myocytes of the pro-hypertensive SHRs have an enhanced cAMP response to ß-adrenergic stimulation; (iii) cross-culturing healthy neurons onto diseased myocytes rescues the myocyte phenotype. Conversely, healthy myocytes can develop a diseased phenotype if diseased neurons are introduced, suggesting the neurons are the dominant drivers behind the sympathetic phenotype in pro-hypertensive states.

The SHRnSHRm co-culture showed significantly elevated myocyte cAMP responses when compared to the WKYnWKYm culture. Interestingly the response to nicotine was consistently elevated in the SHRnSHRm co-cultures, but had a graded appearance in the WKYnWKYm cultures, both reaching a maximum at 100 μM nicotine (Fig. 3e). This could suggest differential sensitivity to nicotine with a potentially larger amount of NA being released per unit of nicotine. This is aligned with the large body of evidence in both human^9^,^35^,^36^ and animal models^13^,^15^,^20^ of hypertension showing increased NA release. It is clear that both the vascular bed and the heart of SHRs have significantly higher expression of nerve growth factor (NGF)^37^. As such, it is conceivable that increased NGF expression could result in increased innervation^25^,^38^ in the SHR animals, since hyper-innervation has been demonstrated in the stroke-prone SHRs both prior to, and after the development of hypertension^39^. This was postulated to be caused by hyperactivity of the stellate ganglion and could help explain the increased incidence of arrhythmias associated with hypertension^40^,^41^.

However, over excitability of the myocytes themselves could also play a part. Indeed, when exposed to increasing concentrations of the ß-adrenergic agonist isoprenaline (0.1–100 nM), the SHR myocytes generated significantly larger cAMP responses when compared to the WKY controls at concentrations ≥ 3 nM (Fig. 2). This is consistent with previous reports demonstrating ß-adrenergic over-activity associated with the hypertensive state^19^,^21^,^22^,^42^,^43^. This was linked to larger basal- and ß-adrenoreceptor stimulated L-type Ca^2+^ currents in pacemaking cells^21^ and ventricular myocytes^22^. The latter may contribute to the increased arrhythmogenic actions of isoprenaline^23^. A study assessing myocardial adrenoreceptors and adenylate cyclase (AC) activity in the developing pro-hypertensive SHRs (0–125 days post birth) demonstrated that whilst ß-adrenergic receptor expression was unchanged between the pro-SHR and WKY, a significant increase in the isoprenaline- and forskolin-induced activity of AC was identified in SHR myocytes^42^. Consistent with that work, a more recent study confirmed these findings and identified significantly increased chronotropic responses of neonatal SHR myocytes to isoprenaline^43^. This could explain the increased isoprenaline-induced cAMP responses of the neonatal SHR myocytes seen in our study. Moreover, plating un-cultured thoracolumbar explants of the SHR onto WKY myocytes supports the notion the myocyte is the dominant driver^2^, although these neurons do not solely innervate the heart and could be contaminated by preganglionic, acetylcholine releasing cells. Interestingly, the increased heart rate responses to isoprenaline are only present in adult animals with established hypertension^19^,^21^, and not in the pro-hypertensive four-week old animals^13^, in contrast to the sympathetic phenotype^17^. Intriguingly, the increased heart rate responsiveness is correlated with the development of increased sympathetic drive^17^ which could suggest that the ß-adrenergic hyper-activity comes about as a result of neuronal dysfunction^10^.

Our results are not consistent with the myocyte being the dominant driver. Given the persistent differences of the myocyte response to isoprenaline at the higher, un-physiological concentrations, one would expect the nicotine-evoked responses of the co-cultures to stay different and not reach a common maximum. Is it possible that a more dominant role is played by the sympathetic neuron, and that the neuronal response to autonomic stimulation takes precedence over any myocyte phenotype? To test this, we developed two cross-cultures; the WKYnSHRm and the SHRnWKYm and exposed them to the same experimental protocol as the WKYnWKYm and SHRnSHRm. To prevent the risk of false negatives due to under-stimulation of the WKYnWKYm co-cultures, and to maximise the chance of resolving the cross-culture phenotypes, 10 μM nicotine was chosen for the cross-culture studies. At this concentration there were clear nicotine evoked cAMP responses from all co-cultures and a significant pro-hypertensive phenotype present in the SHRnSHRm co-cultures. We found that in the WKYnSHRm cross-culture, the normotensive neuron was able to attenuate the elevated cAMP responses in the SHR myocyte, to similar levels to the WKYnWKYm co-cultures (Fig. 5c,d). Moreover, in the SHRnWKYm cross-culture the pro-hypertensive neurons were able to induce a diseased cAMP phenotype in the otherwise healthy WKY myocytes (Fig. 5f,g). Together these results demonstrate that neurons are the principle drivers of post synaptic excitability in this model of cardiac sympathetic hyper-responsiveness associated with the pro-hypertensive state, and support mounting evidence from our laboratory^13^,^15^,^17^ and others^7^,^8^,^9^,^10^,^27^,^44^,^45^,^46^ that neurons may play a more dominant role than previously thought. The precise mechanism whereby the healthy neuron protects the diseased myocyte is not fully established, but is probably related to the neuron releasing less transmitter for a given depolarisation.

What is the molecular mechanism of the neuronal hyper-responsiveness? The data provided here suggest that altered NA release from the sympathetic neuron could account for the differences seen between the WKYnWKYm and SHRnSHRm and its reversal in the cross-cultures since responses were blocked by metoprolol. Indeed, plasma NA levels form an important prognostic marker in the development of hypertension and heart failure^9^,^10^,^28^,^47^ and an indicator of overall mortality^10^. The exact mechanisms behind this also remains unclear, in part due to difficulties in accurately measuring local NA release^9^,^48^,^49^. Early work demonstrated that acetylcholine-induced currents in postganglionic sympathetic neurons had similar properties between the SHR and WKY rats^50^, suggesting that the autonomic phenotype is unlikely to be present at the level of the nicotinic acetylcholine receptor (nAChR). More recent work has demonstrated significantly altered Ca^2+^ handling in the sympathetic neurons of the pro-SHR, manifested in both increased intracellular Ca^2+^ transients^17^ and membrane N-type currents^15^ – both facilitating increased neurotransmission^13^. Emerging evidence suggests oxidative stress leading to dysregulation of cyclic nucleotide signalling plays a major role behind the elevated Ca^2+^. These neurons show impaired nitric oxide (NO) - cGMP signalling resulting in lower cGMP levels and disinhibition of neurotransmission^51^,^52^,^53^,^54^. Moreover we recently reported that the neuronal Ca^2+^ channel phenotype in the SHR is linked to a phosphodiesterase – cyclic nucleotide impairment and failure of cGMP signalling to inhibit I_CaN_^16^.

The neuro-cardiac synapse is a highly specialised zone^28^,^31^,^55^,^56^,^57^ that is structurally dependent on the activity of the sympathetic neurons^31^. The activity-dependence of such processes could have significant relevance to conditions of autonomic imbalance such as hypertension and heart failure since emerging evidence suggests the early hallmarks of the disease appear to present themselves in the nervous system^13^,^15^,^17^. Whether these conditions are associated with molecular remodelling of the synaptic space in the early evolution of the disease process has not been firmly established. In conclusion, our results provide some of the first direct evidence for the dominant role played by the neuron in the initiation and/or maintenance of cardiac sympathetic hyperactivity. We also highlight the potential of developing cell therapies targeted at the post ganglionic, presynaptic neuron to turn down the gain of neurotransmission, akin to a smart pre-synaptic ß-blocker.

## Methods

### Animal models

In this study the spontaneously hypertensive rat (SHR) and its normotensive genetic controls, Wistar Kyoto (WKY) were used, since it has a well established autonomic phenotype at all levels of the cardiac neural axis^13^,^17^,^21^,^39^,^42^,^58^. The SHR begins to show development of clinical symptoms of hypertension from six week of age. All experiments were approved by Oxford University’s Animal Ethics Committee and carried out in accordance with the UK Home Office Animals Scientific Procedures Act, 1986 (PPL 30/3131, David J. Paterson).

### Co-culture of sympathetic stellate neurons and ventricular myocytes

#### Myocyte isolation

Neonatal ventricular myocytes (P3) from SHR and WKY littermates were isolated by enzymatic digestions. Briefly, the hearts were removed and atria discarded to prevent the presence of potentially contaminating intra-cardiac ganglions. The ventricles were cut into ~3 mm^3^ pieces and washed with ice cold Hanks Balanced Salt Solution (HBSS) before being enzymatically digested in a trypsin solution (1 mg/ml in HBSS) rotating at 4 °C for four hours. A series of six, two-minute collagenase (1 mg/ml) digestions in a 37 °C water bath followed. The supernatant from the first round of the collagenase was discarded without disrupting the cells. A further five, two minute incubations in collagenase was completed. After each one, the tissue was titrated 15 times (one/second) using a wide bored (~4 mm diameter) plastic pipette to release single cells. The supernatant containing the cells was placed in cold HBSS before more enzyme was added to the tissues and the digestions continued. The resulting single cell suspension was filtered through a 40 μm filter (EASYstrainer, Greiner bio-one, UK) to remove any cell clusters before the cells were centrifuged at 1000 revolutions per minute for eight minutes to pellet the cells. The pellet was re-suspended in five mL of myocyte plating media and placed into two 35 mm non-coated petri dishes and pre-plated for one hour to remove fibroblasts at 37 °C, 5% CO_2_. Approximately 78 000 cells were plated per four-well plate, each containing four, six mm coverslips coated with Poly-D-Lysine and laminin. This left room for the neurons to sit down in-between the myocytes and achieved a 75% confluent cell layer at day five. The myocyte-only cultures were plated in myocyte media (17% M199, 68% DMEM, 5% fetal bovine serum, 10% horse serum, 1% Penicillin/streptomycin), whilst the myocyte cultures awaiting neurons were plated in co-culture media (myocyte media + 50 ng/ml nerve growth factor, 2.5 S, Millipore).

#### Cardiac sympathetic neuron isolation

The sympathetic stellate neurons were isolated as described in^18^. Briefly, the right and left stellate ganglion were removed from four-week old male pro-hypertensive SHR and normotensive WKY rats. The ganglia were desheathed before being subjected to a series of collagenase (1 mg/ml) and trypsin (2 mg/ml) at 37 °C and triturated to achieve single cell suspension.

#### Co-culture and cross culture formation and nomenclature

Immediately following their culture, the neurons were placed on top of the myocytes to create the normotensive co-culture (WKYnWKYm) and the hypertensive co-culture (SHRnSHRm). The cross-cultures were created by plating WKY neurons on SHR myocytes (WKYnSHRm), and plating SHR neurons on WKY myocytes (SHRnWKYm). The cells were left to settle for 24 hours at 37 °C, 5% CO_2_ before cytosine arabinoside (1 μM) was added to eliminate fibroblast growth. The cultures were maintained for five days to allow synaptic connections to form between the cells^59^. All cultures were exposed to identical experimental protocols and care was taken to ensure the myocytes were innervated by a single neuron. Supplementary video S1 depicts a WKYnWKYm culture on day five post culture.

### Immunofluorescence

Immunofluorescence was performed to confirm myocyte and sympathetic neuron phenotypes and to ensure the absence of cholinergic neurons. Cultures were fixed in 4% paraformaldehyde for 10 minutes before being permeabilised and blocked for one hour using a solution containing 10% goat serum, 0.3% bovine serum albumin and 0.1% Triton X in PBS. Primary antibodies to sarcomeric alpha actinin (myocyte marker, A7811, 1:650) and tyrosine hydroxylase (sympathetic neuron marker, ab152, Abcam, 1:250) and choline acetyl transferase (cholinergic neuron marker, ab144P, Abcam, 1:500) were incubated over night at 4 °C. Alexafluor conjugated antibodies were incubated for two hours before the coverslips were washed and mounted using a DAPI containing soft mount Vectashield (Vectorlabs). Imaging was done using a Live Cell Olympus confocal microscope.

### Imaging of the culture using Förster Resonance Energy Transfer (FRET) imaging

The generation of cAMP in the myocytes in response to receptor activation was assayed using a novel adenoviral cAMP FRET sensor, Ad-Epac-S^H187 16^ as a measure of adrenergic drive in the myocyte.

#### Infection of the FRET sensor

The myocytes were selectively infected with Ad-Epac-S^H187^. 3.6 × 10^5^ particle forming units (PFUs) were added for three hours at 37 °C, 5% CO_2_ before the media was replaced and the cells placed back into the incubator. The cells were left for 24 hours before imaging to allow for adequate expression of the FRET sensor^60^.

#### FRET imaging of cultures

The cultures were perfused with tyrode solution (in mM; 135 NaCl, 4.5 KCl, 11 glucose, 20 HEPES, 1MgCl_2_, 2 CaCl_2_, pH 7.40 - containing drugs when appropriate) at 2.5 ml/min and imaged using an inverted Nikon microscope connected to an OptoLED fluorescence imaging system (Cairn Research Ltd) equipped with a 40x oil-immersion objective, a CoolSnap HQ2 digital CCD camera (Photometics) and a beam-splitter (DV2, Photometrics) which included the emission filters for CFP and YFP acquisition (dichroic mirror 505DCXR). The cells were excited at 430 nm and CFP and YFP emissions were measured as a change in the ratio between 480/535 nm fluorescent emission intensities following a 100 ms excitation at 430 nm, every 15 seconds. Background fluorescence was subtracted from emission intensities and intensity ratios were plotted against time. Mean FRET responses were expressed as the percentage change from baseline (∆R/R0 where ∆R = R-R0. R0 is the ratio of intensity at time = 0 seconds and R is the ratio at time = t seconds)^60^.

#### FRET protocols

The co-cultures were stimulated with different concentrations (1, 10, 100, 300 and 500 μM) of nicotine to physiologically activate the neuron. The cells were perfused with tyrode solution for two minutes to ensure a stable baseline before nicotine was applied. Each culture was exposed to one concentration of nicotine, to prevent confounding results due to the inactivation of the nicotinic receptor. To test whether the responses observed were β_1_ adrenoreceptor mediated, the cultures were pre-incubated with the selective β_1_ receptor antagonist metoprolol (10 μM) for four minutes before nicotine was added. The myocyte-only cultures were exposed to either 0.1, 3 and 30 or 1, 10 and 100 nM isoprenaline to test their sensitivity to β_1_ adrenergic stimulation. Further, they were exposed to 1–500 μM nicotine to ensure nicotine had no non-specific effect on cAMP levels in myocytes cultured alone, and to confirm the absence of any contaminating intra-cardiac neurons.

### Statistical analysis and figure preparation

Data are represented as the mean (±SEM) raw FRET ratios of all data traces. The absolute peak values were expressed as %FRET change from baseline and it was these data the statistical analysis were performed on. All data were found to be normally distributed. Two-way ANOVA with Bonferroni correction was used for the isoprenaline dose-response data in Fig. 2. Unpaired t-tests were used to compare the mean absolute peak values for the nicotine responses in Fig. 3. One-way ANOVA with Bonferroni correction was used to analyse the cross-cultures in Fig. 5. Statistical significance was accepted at p values < 0.05. For each co-culture, 2–3 separate isolations were performed with 6–15 pups. Figures 1b and 5b and e were created using Servier Medical Art according to a Creative Commons Attribution 3.0 Unported License guidelines 3.0 (https://creativecommons.org/licenses/by/3.0/). Simplification and colour changes were made to the original neuron and mycoyte cartoons.

## Additional Information

**How to cite this article**: Larsen, H. E. *et al*. Sympathetic neurons are a powerful driver of myocyte function in cardiovascular disease. *Sci. Rep.* **6**, 38898; doi: 10.1038/srep38898 (2016).

**Publisher's note:** Springer Nature remains neutral with regard to jurisdictional claims in published maps and institutional affiliations.

## Supplementary Material

Supplementary Video S1

Supplementary Information

## References

[b1] Krause T., Lovibond K., Caulfield M. & McCormack T. Management of hypertension: summary of NICE guidance. BMJ 343, d4891 (2011). 21868454 10.1136/bmj.d4891

[b2] Lloyd T. R. & Marvin W. J. Contractile response to sympathetic innervation in neonatal ventricular cardiomyocytes of the spontaneously hypertensive rat. Pediatric Research 30, 207–210 (1991). 1896267 10.1203/00006450-199108000-00016

[b3] Schwartz P. J. Cardiac sympathetic denervation to prevent life-threatening arrhythmias. Nat Rev Cardiol 11, 346–353 (2014). 24614115 10.1038/nrcardio.2014.19

[b4] Tu H. . Heart failure-induced changes of voltage-gated Ca^2+^ channels and cell excitability in rat cardiac postganglionic neurons. American Journal of Physiology - Cell Physiology 306, C132–C142 (2014). 24025863 10.1152/ajpcell.00223.2013 PMC3919990

[b5] Schwartz P. J., La Rovere M. T., De Ferrari G. M. & Mann D. L. Autonomic Modulation for the Management of Patients with Chronic Heart Failure. Circulation: Heart Failure 8, 619 (2015). 25991804 10.1161/CIRCHEARTFAILURE.114.001964

[b6] Ajijola O. A. . Remodeling of stellate ganglion neurons after spatially targeted myocardial infarction: Neuropeptide and morphologic changes. Heart Rhythm 12, 1027–1035 (2015). 25640636 10.1016/j.hrthm.2015.01.045 PMC4411181

[b7] Joyner M. J., Charkoudian N. & Wallin B. G. A sympathetic view of the sympathetic nervous system and human blood pressure regulation. Experimental Physiology 93, 715 (2008). 18326553 10.1113/expphysiol.2007.039545 PMC3433836

[b8] Esler M., Lambert E. & Schlaich M. Point: Chronic activation of the sympathetic nervous system is the dominant contributor to systemic hypertension. J. Appl. Physiol. 109, 1996–8– discussion 2016 (2010). 20185633 10.1152/japplphysiol.00182.2010

[b9] Grassi G., Mark A. & Esler M. The sympathetic nervous system alterations in human hypertension. Circulation Research 116, 976–990 (2015). 25767284 10.1161/CIRCRESAHA.116.303604 PMC4367954

[b10] Mancia G. & Grassi G. The autonomic nervous system and hypertension. Circulation Research 114, 1804–1814 (2014). 24855203 10.1161/CIRCRESAHA.114.302524

[b11] Habecker B. A. . Molecular and cellular neurocardiology: development, and cellular and molecular adaptations to heart disease. The Journal of Physiology 594, 3853–3875 (2016). 27060296 10.1113/JP271840 PMC4945713

[b12] Heaton D. A. . Gene transfer of neuronal nitric oxide synthase into intracardiac ganglia reverses vagal impairment in hypertensive rats. Hypertension 49, 380–388 (2007). 17210833 10.1161/01.HYP.0000255792.97033.f7

[b13] Shanks J. . Cardiac sympathetic dysfunction in the prehypertensive spontaneously hypertensive rat. Am. J. Physiol. Heart Circ. Physiol. 305, H980–6 (2013). 23913706 10.1152/ajpheart.00255.2013 PMC3798753

[b14] Pardini B. J., Lund D. D. & Schmid P. G. Organization of the sympathetic postganglionic innervation of the rat heart. J. Auton. Nerv. Syst. 28, 193–201 (1989). 2628461 10.1016/0165-1838(89)90146-x

[b15] Lu C.-J. . CAPON modulates neuronal calcium handling and cardiac sympathetic neurotransmission during dysautonomia in hypertension. Hypertension 65, 1288–1297 (2015). 25916729 10.1161/HYPERTENSIONAHA.115.05290 PMC4487208

[b16] Larsen H. E., Bardsley E. N., Lefkimmiatis K. & Paterson D. J. Dysregulation of Neuronal Ca^2+^ Channel Linked to Heightened Sympathetic Phenotype in Prohypertensive States. J. Neurosci. 36, 8562–8573 (2016). 27535905 10.1523/JNEUROSCI.1059-16.2016 PMC4987433

[b17] Li D. . Abnormal intracellular calcium homeostasis in sympathetic neurons from young prehypertensive rats. Hypertension 59, 642–649 (2012). 22252398 10.1161/HYPERTENSIONAHA.111.186460 PMC3299568

[b18] Shanks J., Mane S., Ryan R. & Paterson D. J. Ganglion-specific impairment of the norepinephrine transporter in the hypertensive rat. Hypertension 61, 187–193 (2013). 23172922 10.1161/HYPERTENSIONAHA.112.202184

[b19] Herring N., Lee C.-W., Sunderland N., Wright K. & Paterson D. J. Pravastatin normalises peripheral cardiac sympathetic hyperactivity in the spontaneously hypertensive rat. J. Mol. Cell. Cardiol. 50, 99–106 (2011). 20933519 10.1016/j.yjmcc.2010.09.025 PMC3020274

[b20] Zugck C. . Increased cardiac norepinephrine release in spontaneously hypertensive rats: role of presynaptic alpha-2A adrenoceptors. J. Hypertens. 21, 1363–1369 (2003). 12817185 10.1097/00004872-200307000-00026

[b21] Heaton D. A. . Remodeling of the cardiac pacemaker L-type calcium current and its beta-adrenergic responsiveness in hypertension after neuronal NO synthase gene transfer. Hypertension 48, 443–452 (2006). 16847148 10.1161/01.HYP.0000233383.04280.3c

[b22] Xiao Y. F. & McArdle J. J. Elevated density and altered pharmacologic properties of myocardial calcium current of the spontaneously hypertensive rat. J. Hypertens. 12, 783–790 (1994). 7525699

[b23] Barbieri M. . Electrophysiological basis for the enhanced cardiac arrhythmogenic effect of isoprenaline in aged spontaneously hypertensive rats. J. Mol. Cell. Cardiol. 26, 849–860 (1994). 7966353 10.1006/jmcc.1994.1102

[b24] Kreipke R. E. & Birren S. J. Innervating sympathetic neurons regulate heart size and the timing of cardiomyocyte cell cycle withdrawal. The Journal of Physiology 593, 5057–5073 (2015). 26420487 10.1113/JP270917 PMC4667004

[b25] Lockhart S. T., Turrigiano G. G. & Birren S. J. Nerve growth factor modulates synaptic transmission between sympathetic neurons and cardiac myocytes. J. Neurosci. 17, 9573–9582 (1997). 9391012 10.1523/JNEUROSCI.17-24-09573.1997 PMC6573427

[b26] Esler M. Sympathetic nervous system moves toward center stage in cardiovascular medicine: from thomas willis to resistant hypertension. Hypertension 63, e25 (2014). 24420544 10.1161/HYPERTENSIONAHA.113.02439

[b27] Joyner M. J. & Limberg J. K. Blood pressure: return of the sympathetics? Curr. Hypertens. Rep. 18, 7–6 (2016). 26743069 10.1007/s11906-015-0616-3

[b28] Franzoso M., Zaglia T. & Mongillo M. Putting together the clues of the everlasting neuro-cardiac liaison. Biochim. Biophys. Acta, doi: 10.1016/j.bbamcr.2016.01.009 (2016). 26778332

[b29] Ogawa S. . Direct contact between sympathetic neurons and rat cardiac myocytes *in vitro* increases expression of functional calcium channels. J. Clin. Invest. 89, 1085–1093 (1992). 1313444 10.1172/JCI115688 PMC442964

[b30] Qu J., Cohen I. S. & Robinson R. B. Sympathetic innervation alters activation of pacemaker current (If) in rat ventricle. The Journal of Physiology 526, 561–569 (2000). 10922008 10.1111/j.1469-7793.2000.t01-1-00561.x PMC2270045

[b31] Shcherbakova O. G. . Organization of beta-adrenoceptor signaling compartments by sympathetic innervation of cardiac myocytes. J. Cell Biol. 176, 521–533 (2007). 17296797 10.1083/jcb.200604167 PMC2063986

[b32] Ardell J. L. Heart failure: Mechanisms of spinal cord neuromodulation for heart disease. Nat Rev Cardiol (2016). 10.1038/nrcardio.2016.8 26843288

[b33] Kawano H., Okada R. & Yano K. Histological study on the distribution of autonomic nerves in the human heart. Heart Vessels 18, 32–39 (2003). 12644879 10.1007/s003800300005

[b34] Brain K. L., Trout S. J., Jackson V. M., Dass N. & Cunnane T. C. Nicotine induces calcium spikes in single nerve terminal varicosities: a role for intracellular calcium stores. Neuroscience 106, 395–403 (2001). 11566509 10.1016/s0306-4522(01)00280-9

[b35] Esler M., Jennings G. & Lambert G. Noradrenaline release and the pathophysiology of primary human hypertension. Am. J. Hypertens. 2, 140S–146S (1989). 2647104 10.1093/ajh/2.3.140s

[b36] Ferrier C., Cox H. & Esler M. Elevated total body noradrenaline spillover in normotensive members of hypertensive families. Clin. Sci. 84, 225–30 (1993). 8382587 10.1042/cs0840225

[b37] Donohue S. J., Head R. J. & Stitzel R. E. Elevated nerve growth factor levels in young spontaneously hypertensive rats. Hypertension 14, 421–426 (1989). 2676861 10.1161/01.hyp.14.4.421

[b38] Lockhart S. T., Mead J. N., Pisano J. M., Slonimsky J. D. & Birren S. J. Nerve growth factor collaborates with myocyte-derived factors to promote development of presynaptic sites in cultured sympathetic neurons. J. Neurobiol. 42, 460–476 (2000). 10699983

[b39] Kondo M., Fujiwara T. & Tabei R. Noradrenergic hyperinnervation in the heart of stroke-prone spontaneously hypertensive rats (SHRSP). Hypertens. Res. 19, 69–73 (1996). 10968198 10.1291/hypres.19.69

[b40] Fukuda K., Kanazawa H., Aizawa Y., Ardell J. L. & Shivkumar K. Cardiac innervation and sudden cardiac death. Circulation Research 116, 2005–2019 (2015). 26044253 10.1161/CIRCRESAHA.116.304679 PMC4465108

[b41] Vaseghi M. & Shivkumar K. The role of the autonomic nervous system in sudden cardiac death. Progress in Cardiovascular Diseases 50, 404–419 (2008). 18474284 10.1016/j.pcad.2008.01.003 PMC2752648

[b42] Blumenthal S. J., McConnaughey M. M. & Iams S. G. Myocardial adrenergic receptors and adenylate cyclase in the developing spontaneously hypertensive rat. Clin Exp Hypertens A 4, 883–901 (1982). 6807581 10.3109/10641968209060760

[b43] Ohsuzu F. . Enhanced myocardial adenylate cyclase activity in spontaneously hypertensive rats. Jpn. Circ. J. 56, 301–309 (1992). 1313119 10.1253/jcj.56.301

[b44] Esler M. Sympathetic Nervous Activation in Essential Hypertension: Commonly Neglected as a Therapeutic Target, Usually Ignored as a Drug Side Effect. Hypertension 55, 1090 (2010). 20368501 10.1161/HYPERTENSIONAHA.110.151506

[b45] Seravalle G., Mancia G. & Grassi G. Role of the sympathetic nervous system in hypertension and hypertension-related cardiovascular disease. High Blood Press Cardiovasc Prev 21, 89–105 (2014). 24789091 10.1007/s40292-014-0056-1

[b46] Davrath L. R., Goren Y., Pinhas I., Toledo E. & Akselrod S. Early autonomic malfunction in normotensive individuals with a genetic predisposition to essential hypertension. Am. J. Physiol. Heart Circ. Physiol. 285, H1697–704 (2003). 12805027 10.1152/ajpheart.00208.2003

[b47] Buckley U., Shivkumar K. & Ardell J. L. Autonomic regulation therapy in heart failure. Curr Heart Fail Rep 12, 284–293 (2015). 26054327 10.1007/s11897-015-0263-7 PMC4607027

[b48] Grassi G. & Esler M. How to assess sympathetic activity in humans. J. Hypertens. 17, 719–734 (1999). 10459867 10.1097/00004872-199917060-00001

[b49] Seravalle G., Dimitriadis K., Dell’Oro R. & Grassi G. How to assess sympathetic nervous system activity in clinical practice. Curr Clin Pharmacol 8, 182–188 (2013). 23173963 10.2174/1574884711308030003

[b50] Magee J. C. & Schofield G. G. Acetylcholine-induced currents in acutely dissociated sympathetic neurons from adult hypertensive and normotensive rats have similar properties. Pflügers Arch. 429, 772–780 (1995). 7603831 10.1007/BF00374800

[b51] Li D. & Paterson D. J. Cyclic nucleotide regulation of cardiac sympatho‐vagal responsiveness. J Physiol 594, 3993–4008 (2016). 26915722 10.1113/JP271827 PMC4945711

[b52] Li D. . Targeted neuronal nitric oxide synthase transgene delivery into stellate neurons reverses impaired intracellular calcium transients in prehypertensive rats. Hypertension 61, 202–207 (2013). 23172925 10.1161/HYPERTENSIONAHA.111.00105

[b53] Li D. . Efficacy of B-type natriuretic peptide is coupled to phosphodiesterase 2A in cardiac sympathetic neurons. Hypertension 66, 190–198 (2015). 25916722 10.1161/HYPERTENSIONAHA.114.05054 PMC4487831

[b54] Li D., Wang L., Lee C.-W., Dawson T. A. & Paterson D. J. Noradrenergic cell specific gene transfer with neuronal nitric oxide synthase reduces cardiac sympathetic neurotransmission in hypertensive rats. Hypertension 50, 69–74 (2007). 17515453 10.1161/HYPERTENSIONAHA.107.088591

[b55] Landis S. C. Rat sympathetic neurons and cardiac myocytes developing in microcultures: correlation of the fine structure of endings with neurotransmitter function in single neurons. Proc. Natl. Acad. Sci. USA 73, 4220–4224 (1976). 1069311 10.1073/pnas.73.11.4220 PMC431393

[b56] Wingerd K. L. . Alpha 4 integrins and vascular cell adhesion molecule-1 play a role in sympathetic innervation of the heart. J. Neurosci. 22, 10772–10780 (2002). 12486170 10.1523/JNEUROSCI.22-24-10772.2002 PMC6758413

[b57] Birren S. J. & Marder E. Neuroscience. Plasticity in the neurotransmitter repertoire. Science 340, 436–437 (2013). 23620040 10.1126/science.1238518

[b58] Dyke A. C., Angus J. A. & Korner P. I. A functional study of the development of the cardiac sympathetic neuroeffector junction in the SHR. J. Hypertens. 7, 345–353 (1989). 2549123 10.1097/00004872-198905000-00001

[b59] Takeuchi A. . Autonomic nervous system driven cardiomyocytes *in vitro*. Conf Proc IEEE Eng Med Biol Soc 2011, 1945–1948 (2011). 22254713 10.1109/IEMBS.2011.6090549

[b60] Burdyga A. & Lefkimmiatis K. Simultaneous assessment of cAMP signaling events in different cellular compartments using FRET-based reporters. Methods Mol. Biol. 1294, 1–12 (2015). 25783873 10.1007/978-1-4939-2537-7_1

