# Supplementary material for: Sympathetic neurons are a powerful driver of myocyte function in cardiovascular disease
Source: Sci Rep. 2016 Dec 14;6:38898. doi: 10.1038/srep38898 (PMC5155272; doi:10.1038/srep38898)
Supplement: Supplementary Information [file srep38898-s2.pdf]

Supplementary information file for the supplementary video S1 in the following manuscript.

Sympathetic neurons are a powerful driver of myocyte function in cardiovascular disease.

Hege E. Larsen[1,2], Konstantinos Lefkimmiatis[2], David J. Paterson[1,2]*
[1]*OXION Initiative in Ion Channels and Disease*, Sherrington Building, University of Oxford, Oxford OX1 3PT
[2] Burdon Sanderson Cardiac Science Center, Department of Physiology, Anatomy and Genetics, University of Oxford, UK.
* Corresponding author

Supplementary video S1
The supplementary video S1 titled “Real time, phase contrast video of a WKYnWKYm culture on day five post culturing“ was recorded on an Olympus microscope (20x lens) coupled to a Cool Snap CCD camera. The video depicts the real time beating behavior of a co-culture of sympathetic stellate neurons and neonatal ventricular myocytes. The width of the video image is 1 mm. The neuron, whose cell body measures 40 μM is positioned left of the center of the screen.

The full figure legend reads: Real time, phase contrast video of a WKYnWKYm culture on day five post culturing. Note how the sympathetic neuron (middle of the view) branches out and innervates the layer of myocytes.
